# Long-Term Effects of Maternal Deprivation on Redox Regulation in Rat Brain: Involvement of NADPH Oxidase

**DOI:** 10.1155/2017/7390516

**Published:** 2017-03-20

**Authors:** Branka Marković, Nevena V. Radonjić, Gordana Jevtić, Tihomir Stojković, Milica Velimirović, Milan Aksić, Joko Poleksić, Tatjana Nikolić, Dubravka Aleksić, Vidosava Radonjić, Branislav Filipović, Nataša D. Petronijević

**Affiliations:** ^1^Faculty of Sport and Physical Education, University of Belgrade, Blagoja Parovića 156, Belgrade, Serbia; ^2^Department of Psychiatry, University of Connecticut School of Medicine, Farmington, CT, USA; ^3^Institute of Clinical and Medical Biochemistry, School of Medicine, University of Belgrade, Pasterova 2, Belgrade, Serbia; ^4^Institute of Anatomy Niko Miljanić, School of Medicine, University of Belgrade, Dr Subotića 4, Belgrade, Serbia

## Abstract

Maternal deprivation (MD) causes perinatal stress, with subsequent behavioral changes which resemble the symptoms of schizophrenia. The NADPH oxidase is one of the major generators of reactive oxygen species, known to play a role in stress response in different tissues. The aim of this study was to elucidate the long-term effects of MD on the expression of NADPH oxidase subunits (gp91^phox^, p22^phox^, p67^phox^, p47^phox^, and p40^phox^). Activities of cytochrome C oxidase and respiratory chain Complex I, as well as the oxidative stress parameters using appropriate spectrophotometric techniques were analyzed. Nine-day-old Wistar rats were exposed to a 24 h maternal deprivation and sacrificed at young adult age. The structures affected by perinatal stress, cortex, hippocampus, thalamus, and caudate nuclei were investigated. The most prominent findings were increased expressions of gp91^phox^ in the cortex and hippocampus, increased expression of p22^phox^ and p40^phox^, and decreased expression of gp91^phox^, p22^phox^, and p47^phox^ in the caudate nuclei. Complex I activity was increased in all structures except cortex. Content of reduced glutathione was decreased in all sections while region-specific changes of other oxidative stress parameters were found. Our results indicate the presence of long-term redox alterations in MD rats.

## 1. Introduction

Animal models of maternal deprivation (MD) are based on the exposure to stress in early postnatal life. In the majority of mammalians, the mother-infant relation is essential for normal development, and the early loss of maternal care may affect the vulnerability of infants over a lifespan [1]. Early perinatal stress is known to cause various short- and long-term disturbances in cognitive, emotional, and other behavioral performances similar to those seen in schizophrenia [2–4]. Therefore, MD has been proposed as a model of this disease [5–7]. However, little is known about the molecular mechanisms that underlie observed long-term consequences. While the etiology and neuropathology of schizophrenia are still poorly understood, a growing body of evidence indicates that oxidative damage is connected to this disease [8–10]. For a long time, mitochondria have been recognized as the most important source of free radicals but recently the role of NADPH oxidase (NOX) enzymes in redox dysregulation has been suggested [11].

The NOX family is one of the major generators of reactive oxygen species (ROS), which plays an important role in signal transduction. NOX2 is responsible for respiratory oxidative burst in neutrophils, but it is also expressed in the central nervous system, where it controls some of the key neuronal functions and neuroinflammatory processes. The main NOX2-containing cells in the central nervous system are microglia [11, 12]. However, the enzyme is present in other cell types, including neurons. NOX2 is strongly upregulated in different disorders of the central nervous system (CNS), where it generates large amounts of ROS [11, 12]. The knowledge about the role of NOX enzymes in the pathogenesis of schizophrenia is rapidly emerging [12]. Recently, investigations on animal models of schizophrenia involving subchronic administration of subanesthetic doses of ketamine in mice [13] or housing of rats in social isolation [14] have suggested the influence of NOX2-dependent ROS production on cortical circuits.

To the best of our knowledge, no studies have examined the effects of MD on the potential sources of free radicals, especially NOX in the brain. The aim of this study was to investigate the expression of NOX subunits and that of a microglial marker Iba1 (ionized calcium binding adaptor molecule 1) in the different brain regions of adult rats previously subjected to MD on postnatal day nine. We studied the cerebral cortex, hippocampus, thalamus, and caudate nuclei, as these structures have been shown to be affected by MD at the structural and/or functional level [15–18]. The activities of complex I and cytochrome C oxidase (COX), as well as the activities or levels of other oxidative stress parameters were also determined.

## 2. Methods

### 2.1. Animals and Procedures

Male and nulliparous female 3-month-old Wistar rats were obtained from the breeding colony at the Department of Biochemistry, Belgrade University School of Medicine, which originated from Charles River (Charles River Laboratories, Erkrath, Germany). The animals were put together in standard plexiglass cages with sawdust (26 × 42 × 15 cm), in a temperature controlled room (23 ± 1°C) and kept on a standard 12 h light/dark cycle with lights on from 7:00 to 19:00 h, with water and food available ad libitum. Two weeks later, the males were removed and the dams were checked twice daily for delivery. The day of delivery was denoted as postnatal day (P) 0. On P9, eight litters were subjected to maternal deprivation procedure according to the previously published protocols [19, 20]. Briefly, dams were removed from the litter at 10:00 am, after which the pups were weighed and returned to their home cage. They remained in their home cage at room temperature for 24 h. On P10, the pups were weighed again and dams were returned to their cages. The dams of the control litters were removed from their home cages for 3 min and the pups were weighed on both P9 and P10. All litters were later left undisturbed except for the routine cleaning of the cages until P21, when rats were weaned and classified according to sex. Rats were placed in new standard plexiglass cages with sawdust (26 × 42 × 15 cm) and housed in pairs. Rats were sacrificed at 2 months of age (P60). Animals were sacrificed by cervical dislocation and decapitation without anesthesia, to avoid influence of anesthetic drug on measured parameters. All efforts were made to minimize animal suffering and reduce the number of animals used in the study; we used 30 rats overall (15 MD and 15 control group) for various experiments, as indicated below. For biochemical studies only male rats were used in order to avoid sexual dimorphism [4]. All experimental procedures were in full compliance with EU Directive 2010/63/EU and approved by the Ethical Committee of the University of Belgrade.

### 2.2. Quantitative Western Blot Analysis

**Figure 1:**
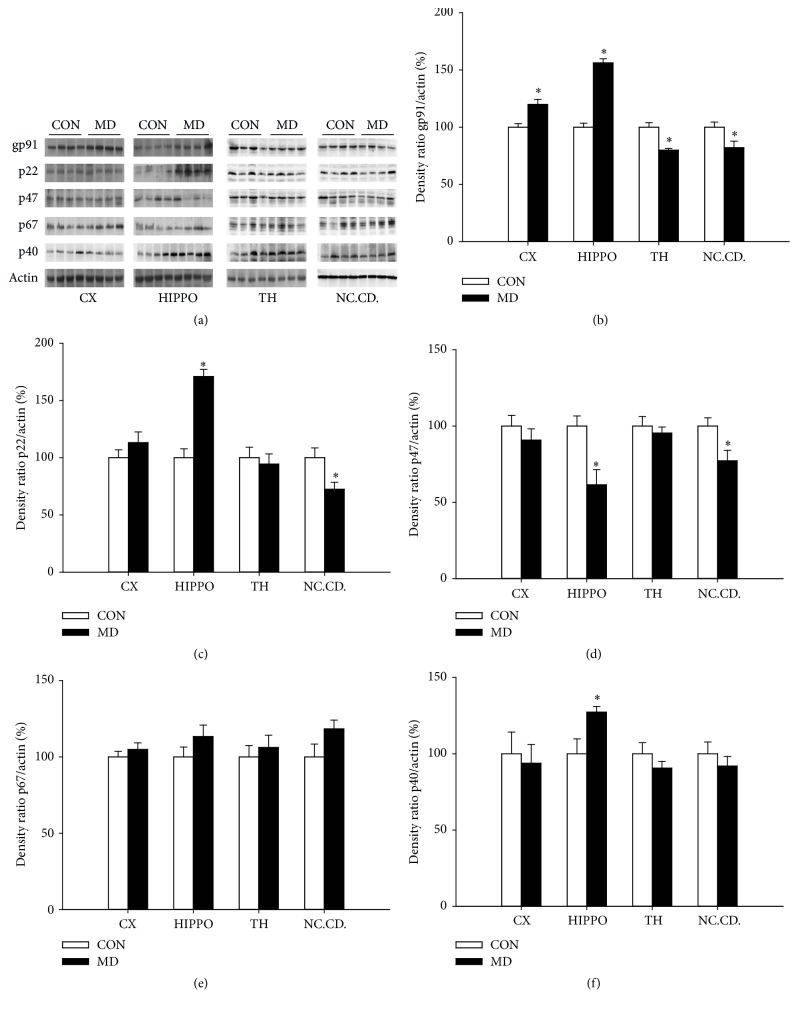
Expression of NADPH oxidase subunits gp91^phox^ (a, b), p22^phox^ (a, c), p47^phox^ (a, d), p67^phox^ (a, e), and p40^phox^ (a, f) in the cortex, hippocampus, thalamus, and nucleus caudatus (P60) of control and MD animals. Results are presented as mean ± SEM; ^*∗*^*p* < 0.05 versus control group; *n* = 4 animals per group.

For Western blot analysis 4 animals from control and 4 from experimental group were sacrificed. Dorsolateral frontal cortex (DlFC, 4.2 mm up to −1.32 mm from bregma), hippocampus, thalamus, and nucleus caudatus were homogenized with an electric homogenizer in lysis buffer [50 mM TrisHCl pH 7.4, 150 mM NaCl, 1% NP-40, 1 mM phenylmethylsulfonyl fluoride, and protease inhibitor cocktail] on ice for 30 min. After centrifugation (18,000*g*) for 15 min at 4°C, supernatants were collected as the cell lysates. Protein concentrations were determined using bovine serum albumin as a standard* (Sigma)* [21]. Equal amounts of protein from each sample (50 *μ*g/well) were separated by SDS-PAGE on 12% gels for approximately 2 hours at 120 V in the running buffer (25 mM Tris base, 192 mM glycine, and 1% SDS). Proteins were transferred to nitrocellulose membranes purchased from Bio-Rad, using the Bio-Rad Transfer System for 70 min. Membranes were blocked with 5% nonfat milk in TBS-T (100 mM Tris base, 150 mM NaCl containing 0.05% Tween-20, pH 7.6) for 60 min. Membranes were incubated with primary antibodies dissolved in TBS-T over night at 4°C. The following primary antibodies were used in this study: gp91^phox^ (1 : 1000, mouse polyclonal,* Santa Cruz, CA*), p22^phox^ (1 : 1000, rabbit polyclonal,* Santa Cruz, CA*), p67^phox^ (1 : 1000, rabbit polyclonal,* Santa Cruz, CA*), gp47^phox^ (1 : 1000, rabbit polyclonal,* Santa Cruz, CA*), gp40^phox^ (1 : 1000, rabbit polyclonal,* Santa Cruz, CA*), anti-SOD1 (1 : 1000, goat polyclonal,* Santa Cruz, CA*), and anti-SOD2 (1 : 1000, goat polyclonal,* Santa Cruz, CA*). Removal of excess primary antibody was carried out by washing the membranes in TBS-T five times for 5 min each. Membranes were incubated with secondary antibody peroxidase-conjugated anti-mouse, anti-rabbit, or anti-goat IgG secondary antibody (anti-mouse, anti-rabbit, and anti-goat,* Southern Biotech, USA*) diluted 1 : 2000 for 1 hour at room temperature. Excess of secondary antibody was removed on the same way as excess of primary antibody. Membranes were exposed to enhanced chemiluminescence reagent (luminol-containing reagent) for 2 min at room temperature and visualized using a ChemiDoc MP system (Bio-Rad). Membranes were stripped and reprobed with anti-*β*-actin antibodies (1 : 10000, mouse monoclonal,* Sigma-Aldrich, USA*) to ensure that all wells were equally loaded. Stripping of membranes was performed by incubation in medium stripping buffer (solution containing glycine, Tris base, and Tween-20, pH 2.2), followed by incubation in phosphate-buffered saline (PBS) and TBS-T. For Figure 1 (detection of NADPH oxidase subunits) membrane for p22 subunit was stripped and incubated with *β*-actin as a control for all subunits, which were all loaded from the same samples at the same time. The protein levels were quantified by densitometry using ImageQuant 5.2 software and expressed relative to *β*-Actin.

### 2.3. Immunohistochemical Analysis

For morphological analysis, 5 male animals from the control and 5 from experimental group were anaesthetized with chloral hydrate (3 mg/kg, i.p.) and transcardially perfused with fixative 4% formaldehyde in 0.1 M phosphate buffer solution (PBS). The brains were postfixed for 24 h at +4°C and cryoprotected by infiltration with sucrose for 2 days at 4°C (20% sucrose in 0.1 M phosphate buffer). Brains were frozen by immersion in 2-methyl-butane (Fluka) precooled to −80°C and stored at −80°C until cutting. Serial coronal sections (25 *μ*m thick) were cut on a cryostat (Leica Instruments, Nußloch Germany). Sections were collected on SuperFrost Plus glass slides (Menzel Braunschweig, Germany) in a standard sequence so that four sections 250 *μ*m apart were present on each slide. Immunofluorescence staining was performed after antigen retrieval (0.01 M sodium citrate solution, pH 9.0, for 30 min at 80°C in a water bath). Nonspecific binding was blocked using 5% normal goat serum, diluted in phosphate-buffered saline (PBS, pH 7.3), and supplemented with 0.2% Triton X-100 and 0.02% sodium azide for 1 h at room temperature (RT). For immunohistochemical studies we used the Iba1 primary antibody (1 : 1500, rabbit polyclonal,* Abcam, UK*). The primary antibody was diluted in PBS (pH 7.3) containing 0.5% lambda carrageenan (Sigma) and 0.2% sodium azide and applied to the sections for 2 days at 4°C. After several washes in PBS, the sections were incubated for 2 h at room temperature with secondary antibody Alexa Fluor 488-conjugated goat anti-rabbit (1 : 200, Invitrogen, A21206) in PBS containing 0.5% lambda carrageenan and 0.2% sodium azide. Following a subsequent wash in PBS, nuclei of the cells were stained with 4,6-diamidino-2-phenylindole (DAPI, 1 : 4000, Molecular Probes, Eugene, USA) for 10 min. Slices were washed in PBS, coverslipped, and allowed to dry for 24 h before analysis. Images of brain sections were obtained on a Axio Imager M2 fluorescent microscope (Carl Zeiss GmbH, Jena, Germany). Stereological counting of Iba1 immunoreactive microglia was performed using a Stereo Investigator software (MicroBirghtField, Williston, VT) according to optical dissector method, as previously described [22]. The parameters for the stereological analysis were as follows: guard space depth, 2 *μ*m; base and height of the dissector, 3600 *μ*m^2^ and 10 *μ*m, respectively; distance between the optical dissectors, 60 *μ*m; objective 40x Plan-Neofluar® 40x/0.75 (Zeiss). Left and right cortical and hippocampal areas were evaluated and results were shown as averaged bilateral values.

### 2.4. Brain Preparation for Measurements of Respiratory Chain Enzyme Activities and Oxidative Stress Parameters

For biochemical analyses 6 animals per group were used. Animals were sacrificed by cervical dislocation and decapitation without anesthesia. After decapitation, the brains were quickly removed and transferred immediately to cold (+4°C) 0.25 M sucrose medium at pH 7.0. Four regions, dorsolateral frontal cortex (dlFC; 4.2 mm up to −1.32 mm from bregma, further refered to as “cortex”), hippocampus, thalamus, and caudate nuclei were directly dissected. Crude synaptosomal fraction for the measurement of oxidative stress parameters and mitochondrial fractions for the measurement of respiratory chain enzyme activities were prepared according to the method of Whittaker and Barker [23]. Isolation of cerebral cortex, hippocampus, thalamus, and nucleus caudatus from individual animals was performed quickly on ice. Isolated tissue was homogenized in ice-cold buffer, pH 7.0, containing 0.25 M sucrose, 0.1 mM EDTA and 50 mM K–Na phosphate buffer. Homogenates were centrifuged twice at 1000 ×g for 15 min at +4°C. The supernatant was further centrifuged at 20,000 ×g for 20 min. Supernatant obtained by this procedure represents crude synaptosomal fraction containing membrane vesicles (microsomes) from smooth and rough endoplasmic reticulum, Golgi and plasma membrane, and all of the soluble components of the cytoplasm. The pellet was resuspended in deionized water and left for 60 min at +4°C. Finally, resuspended pellet was centrifuged at 1700 ×g for 15 min. Obtained supernatant represents crude mitochondrial fraction containing mitochondria, lysosomes, peroxisomes, Golgi membranes, and some rough endoplasmic reticulum.

### 2.5. Determination of Respiratory Chain Enzyme Activity

COX activity was measured by spectrophotometric method of Hess and Pope [24] based on the decrease of absorbance on the 550 nm during the oxidation of ferrocytochrome C to ferricytochrome C. The activity of cytochrome C oxidase was calculated using molar absorption coefficient for reduced and oxidized cytochrome C. Complex I activity was measured according to the method of Janssen et al. [25] using the reaction in which complex I oxidizes NADH and the produced electrons reduce the artificial substrate decylubiquinone that subsequently delivers the electrons to 2,6-dichloroindophenol (DCIP,* Santa Cruz Biotechnology, CA*). The reduction of DCIP is followed spectrophotometrically at 600 nm. All enzyme activities are expressed as units per milligram of protein (U/mg protein).

### 2.6. Determination of Reduced Glutathione

The content of reduced glutathione (GSH) is determined spectrophotometrically using DTNB* (Sigma Aldrich, D8130)*. DTNB reacts with aliphatic thiol compounds at pH 8.0 forming yellow p-nitrophenol anion [26] whose absorption is measured spectrophotometrically at 412 nm. Standard curve was constructed using GSH* (Sigma Aldrich, G4251)* standard solutions in concentrations from 0.1 to 1.0 mM. Results are expressed as nmol per milligram of proteins (nmol/mg protein).

### 2.7. Determination of *γ*-GCL Activity

Determination of *γ*-glutamylcysteine ligase (*γ*-GCL) activity is preformed using the method described by Seelig and Meister [27]. It is based on indirect measurement of ADP formed in a reaction mixture containing L-glutamate, L-*α*-aminobutyrate, and ATP. Concentration of ADP using pyruvate kinase, lactate dehydrogenase, phosphoenol pyruvate, and NADH is measured as a decrease of absorbance at 340 nm enzyme activity is expressed as unit per milligram of protein (U/mg protein).

### 2.8. Determination of Antioxidant Enzyme Activities

The total superoxide dismutase (SOD) activity was assayed as the ability of crude synaptosomal fraction to inhibit the radical-mediated autoxidation of epinephrine [28]. To assay SOD activity, samples were added to 50 mM sodium carbonate buffer at the pH 10.2 and the epinephrine was added at time zero. Activity of SOD was expressed as the percentage of inhibition of the control absorption at 480 nm. SOD purified from bovine blood was purchased from the* Sigma Chemical Company* and used as a standard.

Glutathione peroxidase (GPx) activity was determined on the basis of oxidation of GSH with GPx using NADPH in a reaction catalyzed by enzyme GR. Decrease of absorbance at 340 nm as a result of used NADPH + H^+^ represents the measure of GPx activity in coupled reaction with GR [29]. GR activity is determined by the method of Carlberg and Mannervik [30] based on the reduction of glutathione in the presence of NADPH. NADPH consumption, proportional to the GR activity, is measured at 340 nm. Molar extinction coefficient for NADPH at 340 nm is used for calculations of GPx and GR activities. All enzyme activities are expressed as units per milligram of protein (U/mg protein).

### 2.9. Lipid Peroxide Measurement (MDA)

Thiobarbituric acid after stimulation of lipid peroxidation reacts with malondialdehyde (MDA) released from polyunsaturated fatty acids forming a colored complex whose absorption is measured at 533 nm [31]. Molar extinction coefficient for MDA is used for calculations. Results are expressed as nmol of MDA per milligram of proteins (nmol/mg protein).

### 2.10. Nitrite Measurement

Nitrite (NO_2_^−^) measurement was used as an indirect method for the detection of nitric oxide. Oxidation of nitric oxide generates mostly NO_2_^−^. The concentration of NO_2_^−^ was determined by the Griess reaction, whereby NO_2_^−^ reacts with sulphanilamide in an acidic solution of N-(1-naphthyl)ethylenediamine to give a colored azo product, as previously described [32].

### 2.11. Protein Concentration Determination

Protein concentrations in the samples used for biochemical analysis are determined by the method of Lowry et al. [33] using bovine serum albumin as a standard.

### 2.12. Statistical Analysis

All numerical data are presented as group mean values with standard error of the mean (SEM). As our data were normally distributed, tested by Shapiro-Wilk test, all comparisons were performed using Student's *t*-test. The differences were considered to be significant at values of *p* < 0.05.

## 3. Results

### 3.1. Effects of Maternal Deprivation on the Expression of NADPH Subunits in the Cortex, Hippocampus, Thalamus, and Caudate Nucleus

Quantitative Western blot analysis have revealed increased expression of gp91^phox^ both in the cortex (*p* = 0.0046) and in the hippocampus (*p* = 0.0003) and decreased expression in thalamus (*p* = 0.0035) and caudate nuclei (*p* = 0.0494) of MD group compared to control group (Figures 1(a) and 1(b)). The expression of p22^phox^ was increased (*p* = 0.0004) in the hippocampus of MD group but decreased in caudate nuclei (*p* = 0.0398), while in the cortex (*p* = 0.6783) and thalamus expression it remained unchanged (*p* = 0.4508) compared to control group (Figures 1(a) and 1(c)). Expression of p47^phox^ (Figures 1(a) and 1(d)) was decreased in the hippocampus (*p* = 0.0181) and caudate nuclei (*p* = 0.0399) and unaltered in the cortex (*p* = 0.3999) and thalamus (*p* = 0.5601) of MD group compared to control animals. Expression of p67^phox^ (Figures 1(a) and 1(e)) was unchanged in all investigated structures of MD group compared to control animals (*p* = 0.2311, *p* = 0.2296, *p* = 0.5919, and *p* = 0.1196 for cortex, hippocampus, thalamus, and nucleus caudatus, resp.). Expression of p40^phox^ (Figures 1(a) and 1(f)) was increased in the hippocampus (*p* = 0.0406) and unchanged in other investigated brain structures compared to control group (*p* = 0.7571, *p* = 0.3114, and *p* = 0.4529 for the cortex, thalamus, and caudate nucleus, resp.).

### 3.2. Effects of Maternal Deprivation on the Expression of Iba1 Protein in the Cortex, Hippocampus, Thalamus, and Caudate Nucleus

**Figure 2:**
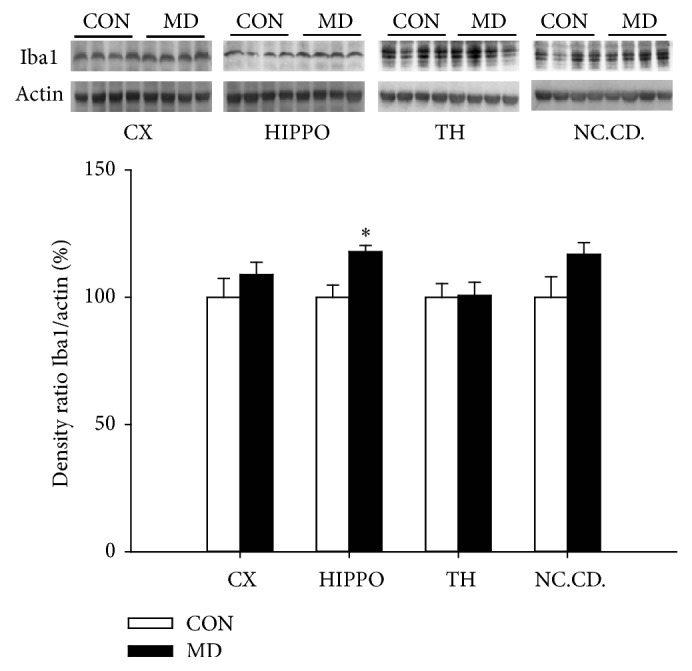
Expression of Iba1 in the cortex, hippocampus, thalamus, and nucleus caudatus in control and MD animals (P60). Results are presented as mean ± SEM; ^*∗*^*p* < 0.05 versus control group; *n* = 4 animals per group.

Quantitative Western blot analysis have revealed increased expression of Iba1 protein (*p* = 0.0177) in the hippocampus of maternally deprived animals, while in the cortex (*p* = 0.4537), thalamus (*p* = 0.9315), and caudate nuclei (*p* = 0.1208) Iba1 expression remained unchanged compared to control group of animals (Figure 2).

**Figure 3:**
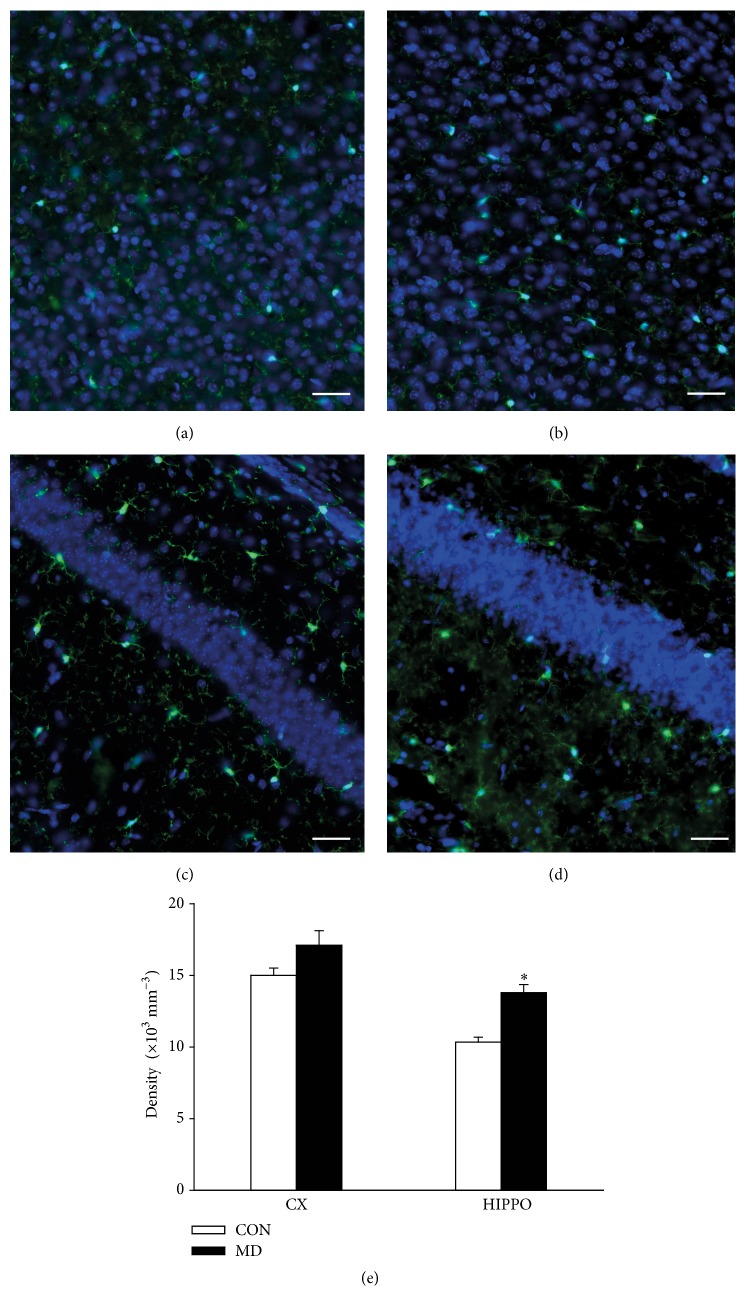
Representative immunohistochemical staining of Iba1 (green) in coronal sections of the cortex (a, b) and hippocampus (c, d) of control (a, c) and MD (b, d) rats; nuclear staining with DAPI (blue). Scale bars: 20 *μ*m. (e) Numerical density of Iba1 immunostained cells in the cortex (CX) and hippocampus (HIPPO). Results are presented as mean ± SEM; ^*∗*^*p* < 0.05 versus control group; *n* = 5 animals per group.

To confirm Western blot results we immunostained brain sections for Iba1. Representative immunofluorescence pictures of Iba1 stainings show a tendency to an increased expression in both the cortex (Figures 3(a) and 3(b)) and hippocampus (Figures 3(c) and 3(d)) of MD rats compared to controls. However, only in the hippocampus the number of Iba1 immunostained cells was significantly increased in MD group (Figure 3(e)).

### 3.3. Effects of Maternal Deprivation on Respiratory Chain Enzyme Activity

Cytochrome C oxidase activity was unchanged in all of the studied structures of MD treated animals compared with controls, while complex I activity was increased in the hippocampus (*p* = 0.00004), thalamus (*p* = 0.0013), and caudate nuclei (*p* = 0.013), while in the cortex (*p* = 0.7171) no change in the complex I activity was observed (Table 1).

### 3.4. Maternal Deprivation Decreased Levels of GSH

**Figure 4:**
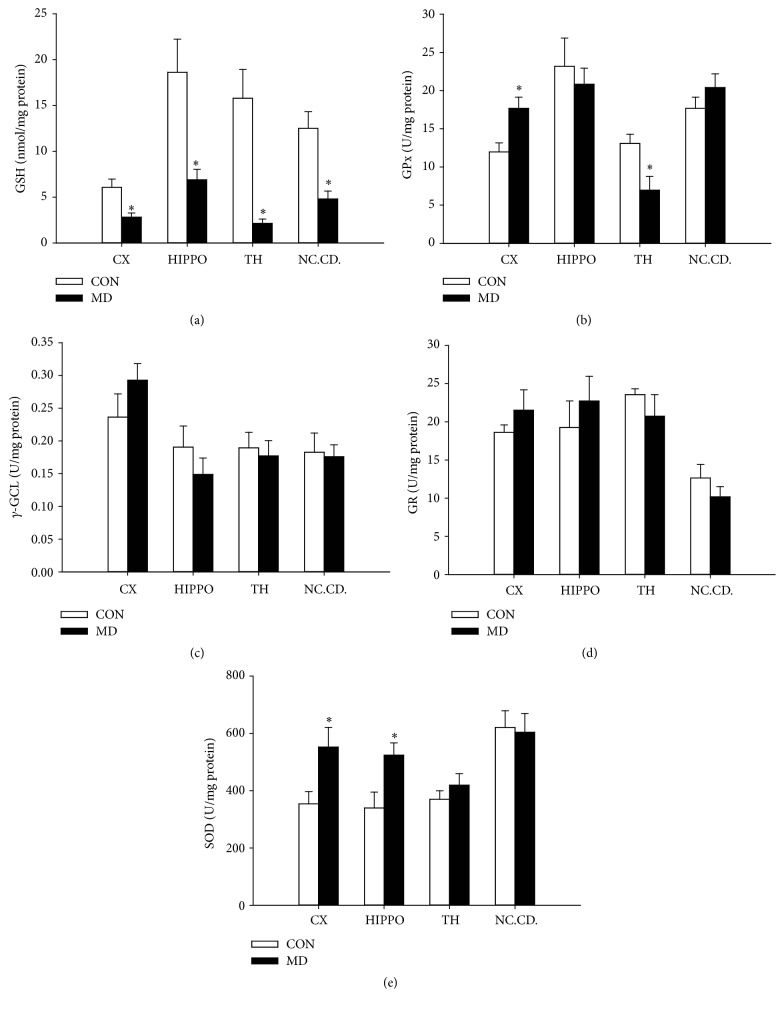
Level of GSH (a), activity of *γ*-GCL (b), activity of GPx (c), and activity of GR (d) in control and MD animals (P60) in synaptosomal fractions of different brain structures. Results are presented as mean ± SEM; ^*∗*^*p* < 0.05 versus control group; *n* = 6 animals per group.

The levels of GSH were significantly decreased in all investigated brain structures: cortex (*p* = 0.0093), hippocampus (*p* = 0.0015), thalamus (*p* = 0.0015), and caudate nucleus (*p* = 0.003) of MD treated animals compared to control group (Figure 4(a)).

### 3.5. Maternal Deprivation Effects on Activities of Enzymes Involved in Glutathione Metabolism

The activity of GPx in the cortex of MD treated animals was increased (*p* = 0.0129), while in the thalamus the activity of this enzyme was decreased (*p* = 0.0188) (Figure 4(b)). In the hippocampus and caudate nuclei no change in the GPx activity was observed (*p* = 0.5958 and *p* = 0.6987, resp.).

There was no significant difference in the activity of *γ*-GCL (Figure 4(c)) and GR (Figure 4(d)) in any of the studied structures of MD treated animals compared with controls.

### 3.6. Effects of Maternal Deprivation on SOD Activity and the Expression of SOD1 and SOD2 in the Cortex, Hippocampus, Thalamus, and Caudate Nucleus

**Figure 5:**
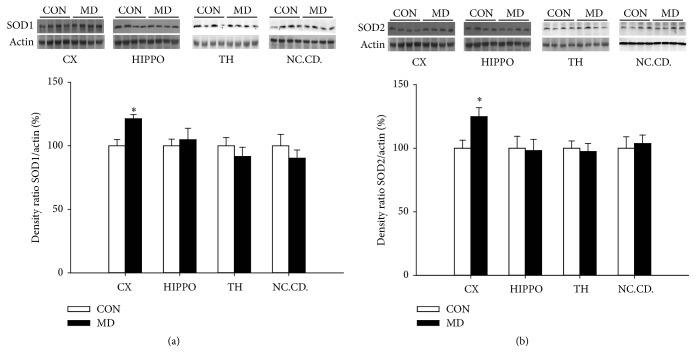
Expression of SOD1 (a) and SOD2 (b) in the cortex, hippocampus, thalamus, and nucleus caudatus in control and MD animals (P60). Results are presented as mean ± SEM; ^*∗*^*p* < 0.05 versus control group; *n* = 4 animals per group.

In the cortex and hippocampus, the activity of SOD was increased (*p* = 0.0331 and *p* = 0.0253) while in the thalamus (*p* = 0.3458) and caudate nuclei (*p* = 0.8536) no changes were observed compared to control group (Figure 4(e)). Quantitative Western blot analysis has revealed increased expression of SOD1 (*p* = 0.0042) and SOD2 (*p* = 0.0219) in the cortex while in the hippocampus, thalamus, and caudate nucleus of MD group no changes were observed compared to control group (*p* = 0.8948, *p* = 0.8548, and *p* = 0.41 for SOD1; and *p* = 0.8548, *p* = 0.7796, and *p* = 0.7481 for SOD2, resp.), see Figures 5(a) and 5(b).

### 3.7. Maternal Deprivation Increases Lipid Peroxidation in the Cortex and Thalamus

Measurement of MDA has shown increased lipid peroxidation in the cortex (*p* = 0.0456) and thalamus (*p* = 0.0172) of MD treated rats, whereas no change was observed in the hippocampus (*p* = 0.9704) and caudate nuclei (*p* = 0.531) compared to controls (Table 2).

### 3.8. Maternal Deprivation Increases Nitrite Concentration in the Cortex and Hippocampus

Measurement of NO_2_^−^, as an indirect indicator for nitric oxide, has shown increased amounts in the cortex (*p* = 0.0101) and hippocampus (*p* = 0.0024) of MD treated rats, whereas no change was observed in the thalamus (*p* = 0.4464) and caudate nuclei (*p* = 0.3467) compared to controls (Table 2).

## 4. Discussion

This study has revealed region-specific changes of the investigated parameters of oxidative stress in MD rats compared to controls. We demonstrated increased expression of membrane NOX2 subunits in the cortex and hippocampus and decreased expression of both membrane and cytosolic subunits in the caudate nucleus. The expression of Iba1 was increased in the hippocampus. Cytochrome C oxidase activity was unaltered in all investigated structures, while complex I activity was increased in all structures except the cortex. Decreased GSH content was observed in all studied brain regions and region-specific differences of the other oxidative stress parameters were detected.

The NOX2 enzymatic complex is composed of two membrane subunits gp91^phox^ (also known as Nox2) and gp22^phox^ and four cytosolic subunits gp67^phox^, gp47^phox^, and gp40^phox^ and Rac [34]. Upon stimulation, the cytosolic subunits translocate to the membrane and form a complex with the membrane-bound components that yields an active enzyme. p67^phox^ and Rac act as the activators of gp91^phox^, whereas p47^phox^ acts as the organizer, ensuring that all subunits are properly aligned for optimal function [35, 36]. The ROS produced by NOX seem to have two general downstream physiological roles. Superoxide produced by NOX2 is required for the respiratory burst that occurs in phagocytes. The second role of NOX is in signaling: O_2_^−^ and hydrogen peroxide (H_2_O_2_) that are derived from NOX enzymes can specifically and reversibly react with proteins, altering their activity, localization, and half-life [37].

In our study, in the cortex, the single change in NOX2 was increased expression of membrane subunit gp91^phox^. On the other hand, in the hippocampus, increased expression of gp91^phox^ was accompanied by increased expression of p22^phox^ and p40^phox^, as well as decreased expression of p47^phox^. Also, the hippocampus was the only structure in which significantly increased expression of microglial marker Iba1 was observed. We interpret the increased Iba1 expression in the hippocampus of MD rats as a sign of possible intensive synaptic remodeling and possibly repair of the damage caused by oxidative stress. We hypothesize that changes in the expression of this microglial activation marker are most prominent in the hippocampus, as the region with the most intensive synaptic plasticity. It is noteworthy that the same trend for the increased Iba1 expression in MD rats was observed in the cortex and caudate nucleus as well, although in these structures it did not reach statistical significance. The changes observed in the cortex indicate that NOX could be an important source of free radicals. On the other hand, the opposite changes in the expression of membrane and regulatory subunits observed in the hippocampus could be explained by the presence of negative feedback that regulates the ROS level in the brain. In nonphagocytic cells, NADPH oxidase generates ROS that are involved in regulation of many cellular activities such as transcription, intracellular signaling, and host defense. Moreover, it has been suggested that NADPH oxidase might be the source of superoxide required for hippocampal long-term potentiation and hippocampus-dependent memory. The expression of synaptically localized NADPH oxidase protein complex has been demonstrated in the mouse hippocampus [38]. The involvement of this enzyme in complex memory processes in the hippocampus requires precise regulation, specifically due to the narrow gap between redox regulation and oxidative damage. NOX2 gene expression is regulated by repressing and activating factors. Repressing and activating factors are involved in many cellular processes including embryonic development and carcinogenesis [34]. Increased expression of Iba1 and increased numbers of microglia in the hippocampus suggest that microglia is the source of increased NOX expression and increased free radical production, as previous studies have implicated involvement of microglia in a production of intracellular [39, 40] and released [41] superoxide.

Perturbations in metabolism are a well-documented, if complex, facet of schizophrenia pathology. Optimal cellular performance requires proper functioning of the electron transport chain, which constitutes four enzymes located within the inner membrane of mitochondria [42]. Ultrastructural changes leading to mitochondrial dysfunction have also been suggested as a key pathway in the pathogenesis of schizophrenia [43, 44].

Our study has shown increased activity of complex I in all structures except in the cortex, without changes of COX activity in any of the investigated brain regions. The increased activity of complex I without concomitant increase in COX activity could lead to the increased direct release of electrons from coenzyme Q to oxygen and formation of free radicals. The impaired cellular respiration and perturbation of mitochondrial network dynamics [45, 46] have been demonstrated as consequences of altered complex I activity.

Mitochondrial dysfunction was also found in the ketamine induced rat model of schizophrenia, where the increased activities of mitochondrial respiratory chain complexes I, II, I–III, and IV were demonstrated in the prefrontal cortex, striatum, and hippocampus in a structure specific manner [47–50].

Results of our study have confirmed the presence of oxidative stress in MD rats. One of the most prominent changes observed in our experiments was a decrease of GSH content in the brain, as a long-lasting effect of early MD. The tripeptide (*γ*-glutamyl-cysteine-glycine) GSH represents the major endogenous antioxidant and redox buffer of the cell and the decrease of its content has frequently been found in patients with schizophrenia and in animal models of this disease. Decreased levels of GSH have been reported in erythrocytes [48–50] and cerebrospinal fluid [51] of untreated schizophrenic patients, as well as in post mortem studies of medicated schizophrenic patients in the prefrontal cortex [52] and caudate region [53]. The investigations on the phencyclidine animal model of schizophrenia [54, 55] have also suggested a decreased GSH concentration as one of the major disturbances in oxidative stress parameters.

The level of GSH in the cell depends of the availability of the precursors and of the activity of GSH replenishing enzymes. However, although the levels of GSH in investigated brain structures in MD rats were markedly decreased, the activity of *γ*-GCL, the first and rate-limiting step in the GSH synthesis, was not changed. This finding suggests an appropriate synthesis of GSH and indicates its conversion into an oxidized form (GSSG). Furthermore, our study has revealed increased activity of GPx only in the cortex of MD rats. This enzyme converts peroxides and hydroxyl radicals in nontoxic forms with concomitant oxidation of GSH into GSSG. Activity of the enzyme GR, which regenerates GSSG into its reduced form (GSH), was also unchanged. The increased activity of GPx and the absence of the adequate increase of GR activity could explain the changes observed in the cortex, but the changes in the activities of GR and GPx in other investigated structures could not account for the marked reduction of the GSH levels observed in these structures. We also detected the increased activity of SOD in the cortex and hippocampus of MD rats. In the cortex, expressions of both cytosolic (SOD1) and mitochondrial (SOD2) isoforms of this enzyme were increased.

Taken together, these findings are indicative for the presence of a redox imbalance in MD rats. However, the oxidative damage, manifested as an increase in MDA concentration, was detected only in the cortex and thalamus. This is consistent with the interpretation that in these structures the production of free radicals has exceeded the defense mechanisms, and the lipid structures were injured. However, our estimation of nitric oxide, based on the NO_2_^−^ measurement, by which we assessed another way of oxidative damage through reactive nitrogen species [56], has shown increased values in the cortex and hippocampus of MD rats. Thus, it may be that various parameters are differentially affected in specific brain structures, indicating even more intricate pattern of oxidative damage upon maternal deprivation.

Findings of structure specific changes in the brain of MD rats in our investigations are in congruence with the findings of Zugno et al. [57]. These authors have investigated the effects of MD conducted for 3 h per day during the first 10 days of life on the oxidative stress parameters in the cortex, hippocampus, and striatum of 60-day-old rats. They demonstrated that MD induces an increase in lipid peroxidation only in the hippocampus. Structure specific changes have often been demonstrated in schizophrenia [58]. The reason for the increased sensitivity of specific brain structures after stressful events could lie in the fact that stress alters dopamine release in the prefrontal cortex and striatum, and these changes are mediated by the NMDA receptors [59]. Our finding of the decreased expression of NOX2 subunits in the striatum may be indicative for decreased capacity of this structure to cope with the challenging conditions that need appropriate redox regulation.

Several studies have investigated the acute influence of MD on oxidative stress in the brain, but there are no results of the long term effects of this perinatal procedure. Uysal et al. [60] have investigated immediate effects of MD on the oxidative stress. These authors have shown that changes in lipid peroxidation and antioxidant enzymes activities in the brain depend of the age and sex of rats subjected to MD. Early postnatal maternal deprivation on P6 for 24 hours caused a significant decrease in brain lipid peroxidation and a significant increase in GPx activity in the hippocampus, prefrontal cortex, and striatum, as an immediate response, that is, on P7. In addition, although an increase in SOD enzyme activity and lipid peroxidation in the brain of maternally separated male rats was observed, these parameters were stable in female rats suggesting that female hormones are protective, possibly through a scavenging action of estrogens [61]. In congruence with our study are the results of Diehl et al. [62], which revealed that periodic maternal separation, for three hours daily during the first ten days of life, leads to an increase in the oxidative damage manifested as increases in DNA breaks in the three-month-old animals.

Excessive ROS production can lead to oxidative death in neurons. Our previous study [63] demonstrated that MD has a long-term effect on brain morphology causing a decrease in hippocampal volume, as well as a decreased thickness of the prefrontal, retrosplenial, and motor cortex in rats' brain and decreased expression of NeuN, a neuronal marker, which now can be related to excessive production of ROS and disturbed oxidative defense demonstrated in the present study.

Our results are indicative for the involvement of NOX in the development of long-lasting effects of early maternal deprivation. Further investigations are necessary in order to fully elucidate the exact mechanisms behind this connection and eventually use the obtained knowledge in revealing new therapeutic strategies.

## 5. Conclusion

Early maternal deprivation produces long-term redox alterations in the brain of rats. In the cortex, NOX seems to be the main source of free radicals. In the hippocampus, both NOX and respiratory chain disturbances could contribute, while in the other brain structures an altered respiratory chain is probably the primary cause of redox imbalance.

## Figures and Tables

**Table 1:** Complex I activity and cytochrome C oxidase activity in mitochondrial fraction of cortex, hippocampus, thalamus, and nucleus caudatus in control and MD group of animals (P60). Results are presented as mean ± SEM; ^*∗*^*p* < 0.05 versus control group; *n* = 6 animals per group.

	Complex I (U/mg protein)		COX (U/mg protein)	
	Control	MD	Control	MD
Cortex	186.3 ± 6.2	194.7 ± 21.8	11.2 ± 0.8	10.0 ± 0.7
Hippocampus	43.4 ± 7.1	136.6 ± 12.0^*∗*^	3.89 ± 0.8	3.69 ± 0.4
Thalamus	80.4 ± 9.6	178.2 ± 20.1^*∗*^	3.73 ± 0.8	3.48 ± 0.7
Nc.Caudatus	49.9 ± 7.3	95.3 ± 7.2^*∗*^	2.4 ± 0.4	2.82 ± 0.4

**Table 2:** Levels of MDA and NO_2_^−^ in control and MD group of animals (P60) in synaptosomal fraction in different brain structures. Results are presented as mean ± SEM; ^*∗*^*p* < 0.05 versus control group; *n* = 6 animals per group.

	MDA (nmol/mg protein)		NO_2_^−^ (nmol/mg protein)	
	Control	MD	Control	MD
Cortex	32.0 ± 3.2	44.0 ± 4.2^*∗*^	21.1 ± 4.3	48.0 ± 7.8^*∗*^
Hippocampus	66.0 ± 8.8	66.5 ± 7.0	34.1 ± 6.0	87.7 ± 12.8^*∗*^
Thalamus	57.4 ± 2.8	82.6 ± 8.4^*∗*^	23.2 ± 9.9	14.6 ± 4.3
Nc.Caudatus	73.8 ± 3.6	80.9 ± 10.4	14.4 ± 4.6	21.7 ± 6.9
